# Comprehensively Analyzed Macrophage-Regulated Genes Indicate That PSMA2 Promotes Colorectal Cancer Progression

**DOI:** 10.3389/fonc.2020.618902

**Published:** 2021-01-18

**Authors:** Jingbo Qi, Zhiqiu Hu, Shaoqun Liu, Fan Li, Sheng Wang, Wuqing Wang, Xia Sheng, Li Feng

**Affiliations:** ^1^ Endoscopy Center, Minhang Hospital, Fudan University and Institutes of Biomedical Sciences, Fudan University, Shanghai, China; ^2^ Department of Surgical, Minhang Hospital, Fudan University, Shanghai, China; ^3^ Endoscopy Center, Minhang Hospital, Fudan University, Shanghai, China; ^4^ Department of Pathology, Minhang Hospital, Fudan University, Shanghai, China

**Keywords:** colorectal cancer, tumor-associated macrophages (TAM), macrophage, PSMA2, cell proliferation, cell metastasis, MiR-132

## Abstract

Colorectal cancer (CRC) is the third most common cancer worldwide. Here, we identified tumor-associated macrophages (TAMs) as regulators of genes in CRC. In total, the expressions of 457 genes were dysregulated after TAM coculture; specifically, 344 genes were up-regulated, and 113 genes were down-regulated. Bioinformatic analysis implied that these TAM-related genes were associated with regulation of the processes of macromolecule metabolism, apoptosis, cell death, programmed cell death, and the response to stress. To further uncover the interplay among these proteins, we constructed a PPI network; 15 key regulators were identified in CRC, including VEGFA, FN1, JUN, CDH1, MAPK8, and FOS. Among the identified genes, we focused on PSMA2 and conducted loss-of-function experiments to validate the functions of PSMA2 in CRC. To further determine the mechanism by which PSMA2 affected CRC, we conducted multiple assays in CRC cell lines and tissues. PSMA2 enhanced the proliferation, migration and invasion of CRC cells. Moreover, our data indicated that PSMA2 expression was dramatically increased in stage 1, stage 2, stage 3, and stage 4 CRC samples. Our data indicated that PSMA2 was one target of miR-132. A miR-132 mimic greatly hindered CRC cell proliferation. In addition, the luciferase assay results revealed that miR-132 directly regulated PSMA2. Moreover, our data indicated that miR-132 expression was greatly decreased in CRC samples, which was associated with longer survival times of CRC patients, implying that miR-132 was a probable biomarker for CRC. Collectively, these data indicate that PSMA2 is a promising target for the therapy of CRC.

## Background

Colorectal cancer (CRC) is regarded as the third most common cancer worldwide, with greatly increasing rates of occurrence and death in China (1, 2). Multiple complex genetic and epigenetic alterations are involved in the progression of CRC (3, 4). For example, FXR modulates the proliferation of intestinal cancer stem cells (5). In colorectal carcinoma, METTL3 facilitates the progression of tumors through an m^6^A-IGF2BP2-dependent mechanism (6). Recently, non-coding RNAs were reported to exert major effects on CRC (7, 8). For example, colorectal carcinogenesis and glucose metabolism were found to be induced by the long non-coding RNA (lncRNA) GLCC1 *via* stabilization of c-Myc (9). The lncRNA SATB2-AS1 modulates SATB2 and then impedes tumor metastasis, thus affecting the microenvironment of tumor immune cells in CRC (10). Thus, it is urgent to establish a more accurate prognostic model for CRC and explore the factors driving tumor initiation.

As immune cells, tumor-associated macrophages (TAMs) usually perform important functions in promoting the growth of tumor cells in the TME by repressing the T cell-induced immune response (10–12). The TME directly mediates tumor growth *via* its interaction with tumor cells (13). TAMs either directly promote tumor cell growth by inducing angiogenesis (14) or indirectly inducing interactions among immune cells in the TME at the primary tumor site (15). TAMs can lead to tumor development through various mechanisms (16). Some studies have implied that an increase in the number of infiltrating macrophages in temporomandibular arthritis is associated with an increased survival rate of CRC patients (17). Sickert et al. and Zhongshan et al. reported that in CRC patients, a reduced number of macrophages were related to an advanced stage of CRC (18). A decrease in the number of CD68^+^ macrophages resulted from different isoforms of VEGF that were responsible for inducing tumor angiogenesis (19, 20). Several reports have shown that secreted genes participate in the regulation of TAMs during CRC progression. For instance, TAMs secrete VEGF to promote CRC angiogenesis and metastasis (21). Secreted VEGF can stimulate NF-*κ*B activation and induce tumor cells to produce IL-10 in a manner mediated by STAT3 (22). In addition, TAMs are the main component of the tumor microenvironment and are usually associated with tumor metastasis in human cancers. Studies have shown that in CRC animal models, macrophage infiltration induced by lipopolysaccharide (LPS) or high cholesterol diet (HCD) significantly promotes the growth of CRC. LncRNA RPPH1 promotes the metastasis of CRC by interacting with TUBB3 and promoting the exosome-mediated polarization of macrophages M2. Exploring the mechanism of TAMs in CRC growth and metastasis could offer new strategies for the treatment of CRC.

It is well known that inhibition of the proteasome is more toxic in cancer cells than in normal cells (23). Another proteasome subunit gene, PSMD 10, also named Gankyrin or P28, was reported to have proteasome-independent biological functions. PSMD 10 acts as an oncogene by increasing Rb hyperphosphorylation by CDK4 and inducing p53 degradation by MDM2 (23, 24). PSMD2 modulates cell proliferation and cell cycle progression in breast cancer by modulating proteasomal degradation of p21 and p27 (23, 25). PSMC2 is highly up-regulated in pancreatic cancer and induces the proliferation of cancer cells, but blocks apoptosis (24). PSMA2 is widely expressed and encodes a peptidase, a component of the *α* subunit of the 20S key proteasome complex (26). The proteasome, a complex of multicatalytic proteases, is widely distributed in eukaryotic cells and degrades peptides in an ATP/ubiquitin-dependent manner (27). PSMA2 plays an important role in controlling many cellular activities, such as cell cycle progression (27, 28). However, the exact function of PSMA2 in CRC is unclear (29).

MiRNA is a small non-coding RNA molecule that contains about 22 nucleotides and plays a role in RNA silencing and post-transcriptional regulation of gene expression. By acting as a tumor suppressor and oncogene, the dysregulation of miRNA expression is associated with various cancers. Non-coding RNA plays a key role in the post-transcriptional regulation of mRNA translation and renewal in eukaryotes. miRNA interacts with its target RNA especially through protein-mediated sequence-specific binding, resulting in an extended and highly heterogeneous miRNA-RNA interaction network. In such a network, the competition for binding miRNAs can generate effective positive coupling between its targets. miRNA works mainly by binding to the 3′untranslated region (3′ UTR) of target mRNA. However, it is not clear how miR-132 mediates PSMA2 to regulate the occurrence and development of CRC.

Here, experiments with CRC tissues and cells (HCT-116 and RKO) were performed to explore the transcriptional level of PSMA2. The specific impact of PSMA2 on CRC proliferation and the antagonistic interaction between PSMA2 and miR-132 were validated. In addition, PSMA2/miR-132 signaling pathway-influenced CRC growth was investigated. The current results provide innovative insight into the function of PSMA2 in CRC occurrence and development. In the future, PSMA2 may potentially be a valuable clinical biomarker in CRC.

## Materials and Methods

### GEO Datasets Analysis

GSE21510 (30), a set of gene expression profile data for CRC, was obtained from the GEO database. All datasets included in the database were composed of more than 10 samples. To obtain the matrix data of each GEO dataset, we performed normalization and log2 transformation. To filter the DEGs in tumor and control tissues, we used the Limma package in R. To integrate the DEGs from the six datasets, we used the RobustRankAggreg (RRA) and limma packages on the basis of a robust rank aggregation method (31). A |log2FC | of >1.5 and an adjusted *P* value of <0.05 were considered the criteria for filtering DEGs with significant differences.

### Functional Enrichment Analysis

The Database for Annotation, Visualization and Integrated Discovery (DAVID, http://david.abcc.ncifcrf.gov/) (32), facilitated the translation of collected data to biological analysis. The DAVID online tool was used to perform Gene Ontology (GO) and Kyoto Encyclopedia of Genes and Genomes (KEGG) pathway enrichment analyses (33). *P*  < 0.01 was considered the cut-off criterion.

### Collection of Tissue

Thirteen paired CRC tissues and paired adjacent normal tissues of patients undergoing colorectal surgery were obtained from Minhang District Central Hospital (Shanghai, China). After collection, all tissues were immediately frozen in liquid nitrogen and then preserved in an ultralow temperature freezer. The Ethics Committee of Minhang Hospital of Fudan University reviewed, approved and supervised the protocols for all experiments utilizing tissues from human patients, and informed consent forms were obtained from all participants.

### Cell Culture and Transfection

HFC, a normal human colorectal cell line provided by Kerafast (ECA001, USA), and two human colorectal cancer cell lines, RKO and HCT-116, provided by ATCC (USA), were seeded in DMEM (Invitrogen) supplemented with 10% FBS (Gibco) and grown in a 37°C incubator with 5% CO_2_. Si-PSMA2 purchased from GeneCopoeia (China) was transfected into the indicated cells to ablate PSMA2. A miR-132 mimic or miR-132 inhibitor (GeneCopoeia) was transfected into cells to overexpress or suppress miR-132. Accordingly, the si-negative control (si-NC) vector, mimic NC, or inhibitor NC was transfected as the negative controls (GeneCopoeia). The sequences are listed as follows: miR-132 mimic (5′-ACCGUGGCUUUCGAUUGUUACU-3′), mimic NC (5′-CAGGUAAUCAACGCGGAGGUCA-3′), miR-132 inhibitor (5′-AGUAACAAUCGAAAGCCACGGU-3′) and inhibitor NC (5′-CGUGGUGCUCGUGAAGGGUCGG-3′), siPSMA2 (5′-CCATTCATACAGCCATCTT-3′). Prior to transfection with the indicated siRNAs, mimics, or inhibitors, cells were inoculated in 96-well or 6-well plates. Cells were harvested 24 h post incubation.

### Extraction and Quantitation of RNA

TRIzol reagent (Invitrogen) was applied to isolate total RNA from cells. To quantify miR-132, we used a Hairpin-it TM miRNA quantitative qPCR kit (GenePharma). The relative gene expression levels of PSMA2 were measured by SYBR Green-based qPCR. GAPDH was utilized as the normalization control. Standard quantification of gene expression levels was conducted with the 2^−ΔΔCT^ method.

### CCK-8 Assay

A CCK-8 assay was applied to measure cell viability. CRC cells transfected with the indicated vectors were reseeded at 1 × 10^3^ cells per well in 96-well plates. On days 1, 2, 3, 4, and 5 post plating, cells were harvested for viability measurements. A colored formazan product that was enzymatically converted from CCK-8 was quantified as a readout for mitochondrial dehydrogenase activity. Briefly, a suitable volume of CCK-8 reagent was pipetted into each sample. After 2 h of incubation at 37°C, the cell numbers were determined by measuring the absorbance at 450 nm using a microplate reader (Bio-Tek).

### Colony Formation

Colony formation test cells were seeded in a 6-well plate (300 cells per well) and incubated at 37°C for 14 days. Colony scoring: Cells were fixed in 0.5 ml methanol for 30 min and stained with crystal violet for 15 min (Beyotime Biotechnology, Nantong, China). The number of colonies was counted in at least three independent experiments, expressed as mean ± SEM.

### Migration and Invasion Assay

The invasion assay was performed in Transwell chambers with membrane filter inserts that were coated with Matrigel (Corning Costar). The same protocols without Matrigel were applied for the migration assay. The chambers were placed in a 24-well plate. Serum-free DMEM was loaded into the upper chambers of Transwell filters. DMEM supplemented with 20% FBS was added to the lower chambers. Cells were only incubated in the upper chambers. After 24 h, cells on the top side of the filter were collected, and the cells that had crossed the membrane were fixed with methanol and stained with DAPI solution (Vazyme, China).

### Luciferase Reporter Assay

A Dual Luciferase Reporter Assay System (Promega, WI, USA) was used to assess relative luciferase gene expression according to the manufacturer’s instructions. Briefly, CRC cells were transfected with wild-type/mutant PSMA2 (wt-PSMA2/mut-PSMA2) plasmids containing the luciferase gene and miR-132 inhibitor or mimic. Forty-eight hours post transfection, we detected relative luciferase reporter gene expression at least three times.

### Statistical Analysis

Our data are shown as the mean ± standard deviation (SD) values. Comparisons between two groups were analyzed by paired Student’s t-test. One-way ANOVA was applied to analyze the differences among more than two groups. All experiments were performed in triplicate, and data analysis was conducted with SPSS 17.0 (34) (USA), with *P* < 0.05 suggesting a statistically significant difference.

## Results

### DEG Identification After Tumor-Associated Macrophage Treatment in Colorectal Cancer

**Figure 1:**
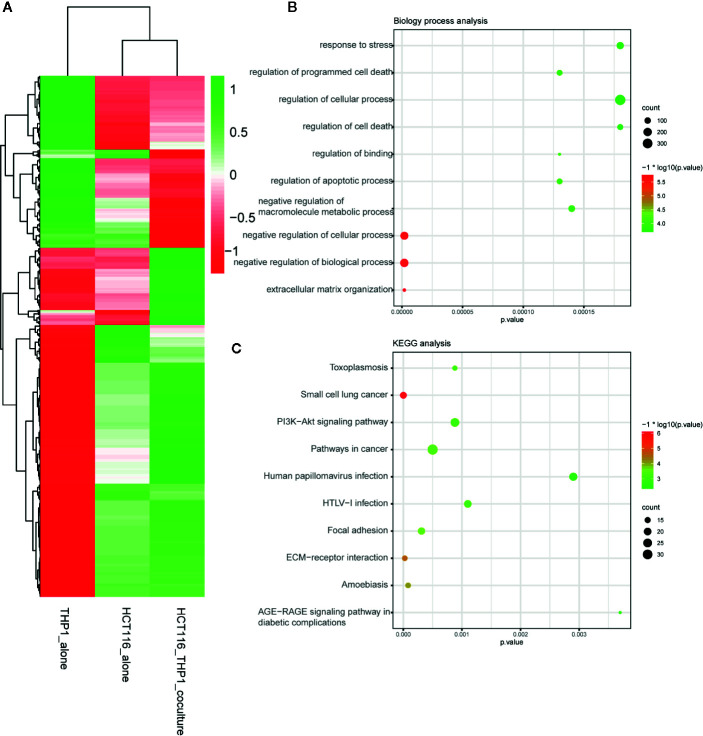
DEG identification after TAM treatment in CRC. **(A)** Demonstrated the increased and decreased genes after the integrated analysis. **(B)** GO analysis of DEG after TAM treatment in CRC. **(C)** KEGG analysis of DEG after TAM treatment in CRC.

A total of 344 up-regulated genes and 113 down-regulated genes among the 457 DEGs were identified by screening with the Limma/RobustRankAggreg packages and integration with the RRA package. **Figure 1A** showed the up-regulated and down-regulated genes identified by the integrated analysis.

### Functional Enrichment Analysis

To explain the biological functions of the 457 genes differentially expressed after TAM treatment in HCT116 cells, we performed biological process and pathway enrichment analyses. The biological processes primarily involved organization of the extracellular matrix, negative modulation of biological processes and cellular processes, macromolecule metabolic processes, apoptotic process regulation, binding regulation, cell death and cellular processes, as well as programmed cell death regulation and the response to stress (**Figure 1B**).

KEGG pathway analysis data indicated that these selected genes primarily participated in AGE-RAGE signaling pathway modulation in diabetic complications, amoebiasis, ECM–receptor interactions, focal adhesion, HTLV-I infection, human papillomavirus infection, cancer pathways, the PI3K–Akt signaling pathway, small cell lung cancer, and toxoplasmosis (**Figure 1C**).

### Hub Genes Selection and Analysis

**Figure 2:**
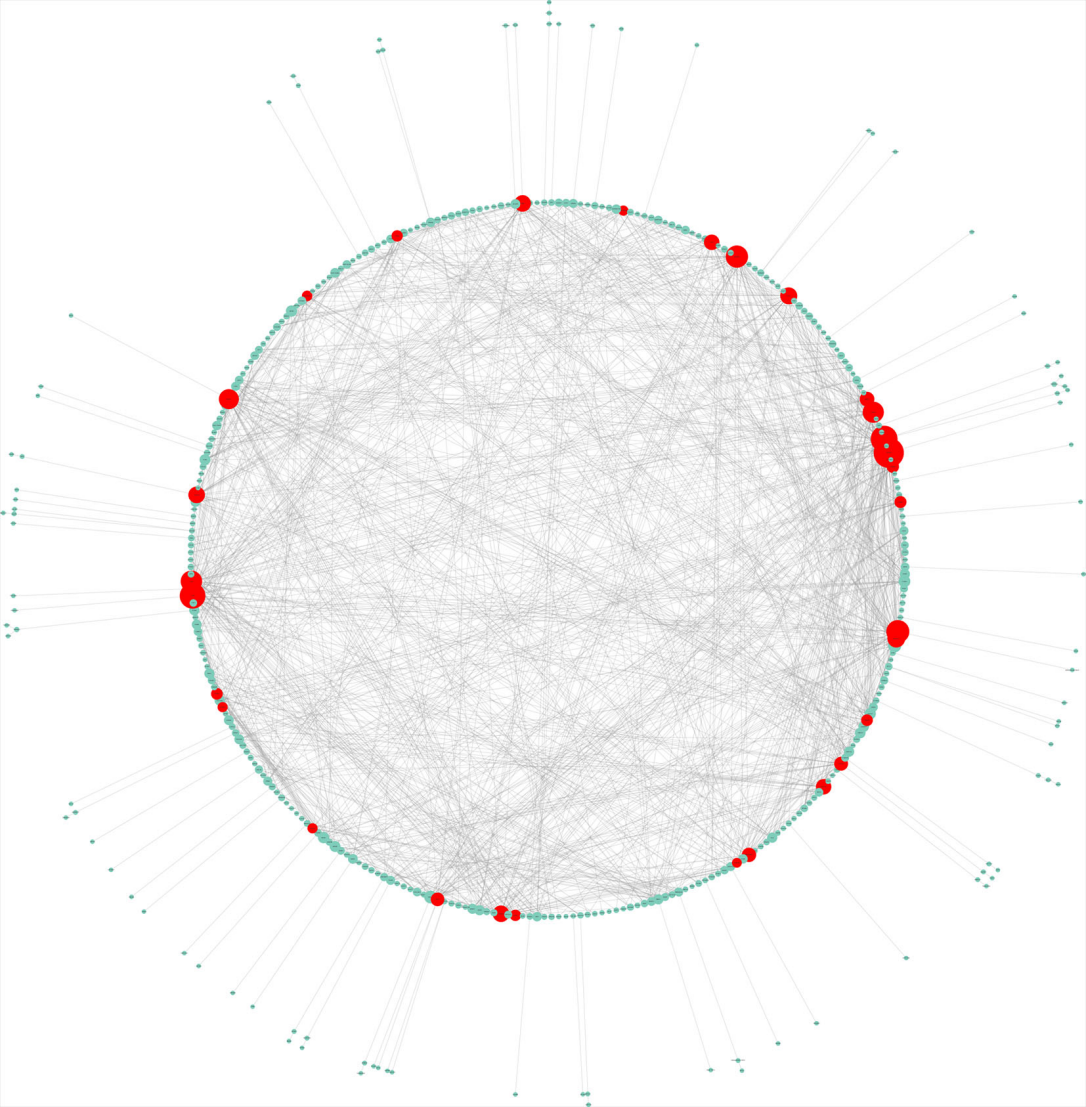
Hub genes selection and analysis. The PPI network of DEGs comprised 334 nodes and 1,568 edges.

The PPI network of DEGs comprised 334 nodes and 1,568 edges. Fifteen genes, namely, VEGFA, FN1, JUN, CDH1, MAPK8, FOS, CXCL8, EGR1, CDKN1A, PLK1, HSPA5, ITGB1, PPARG, ATF3, and COL1A1, were chosen as hub genes and aggregated together in a module in accordance with the cut-off criterion of a degree ≥ 15 (**Figure 2**).

### PSMA2 Promoted Colorectal Cancer Cell Proliferation

**Figure 3:**
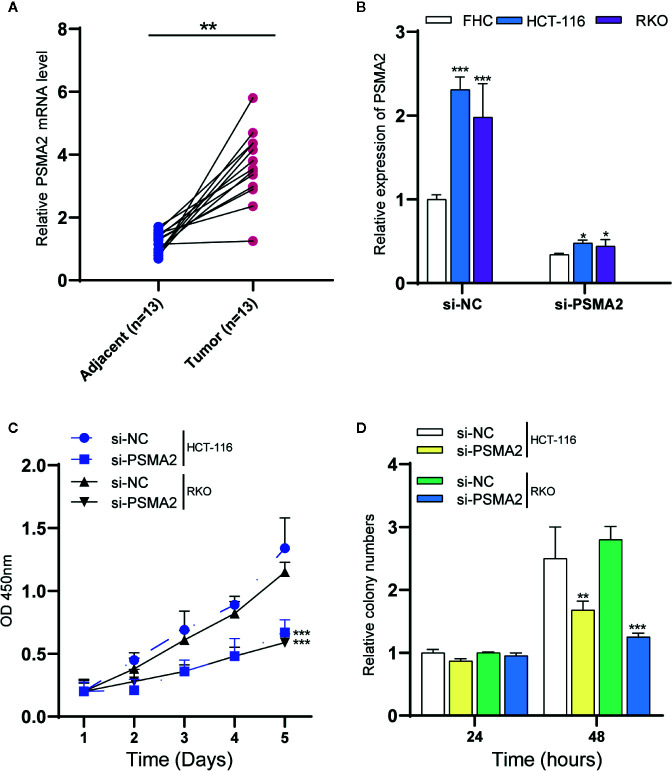
PSMA2 promotes CRC cell proliferation. **(A)** PSMA2 mRNA is up-regulated in CRC samples using clinical samples. **(B)** Transfecting with si-PSMA2 significantly reduced the PSMA2 expression in FHC and two colorectal cancer cell lines. **(C)** PSMA2 silencing declined RKO and HCT-116 cell proliferation using CCK-8 assay. **(D)** PSMA2 silencing suppressed the colony formation abilities of RKO and HCT-116 cell. *P < 0.05, **P < 0.01, ***P < 0.001.

In this study, we focused on exploring the roles of PSMA2, which was connected to 14 different mRNAs, suggesting that it may have a regulatory role in CRC. However, the detailed roles of this gene in CRC remained largely unclear. First, we assessed the PSMA2 expression profile in human CRC tissues. Our data suggested that compared to normal tissues, CRC tissues had high expression of PSMA2 (**Figure 3A**).

RKO and HCT-116 cells were used to evaluate certain effects of PSMA2 on CRC cell proliferation. Si-PSMA2 was separately transfected into the cells indicated above, and the data indicated that the silencing efficiency of PSMA2 was related to the amount of si-PSMA2 transfected to some extent (**Figure 3B**). The qPCR data showed that the PSMA2 level in CRC cell lines was increased when compared to that in the normal cell line FHC. Transfection with si-PSMA2 significantly reduced PSMA2 expression in FHC cells and the two colorectal cancer cell lines. Then, the proliferation of RKO and HCT-116 cells was assessed with CCK-8 assays. As expected, PSMA2 silencing decreased RKO and HCT-116 cell proliferation. Moreover, we found that PSMA2 silencing suppressed the colony-forming ability of RKO and HCT-116 cells (**Figures 3C, D**). These results revealed that CRC cell growth was inhibited by PSMA2 silencing.

### PSMA2 Promoted Colorectal Cancer Cell Migration and Invasion

**Figure 4:**
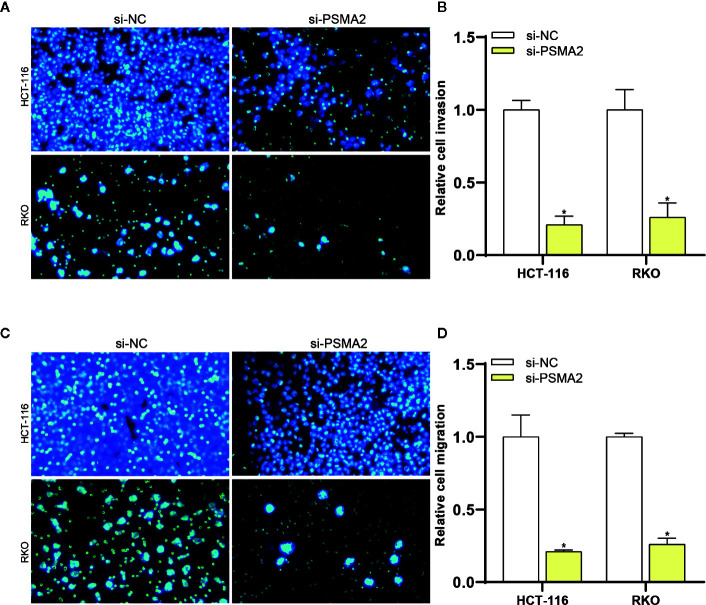
PSMA2 promotes CRC cell migration and invasion. **(A, B)** The migratory capacity of RKO or HCT-116 cells was inhibited after silencing PSMA2. **(C, D)** The invasive capacity of RKO or HCT-116 cells was inhibited by PSMA2 silencing. *P < 0.05.

Then, the effect of PSMA2 on invasion was assessed by a Transwell assay. As shown in **Figures 4A, B**, the invasive capacity of RKO and HCT-116 cells was reduced after PSMA2 silencing. Then, the effect of PSMA2 on migration was investigated by a Matrigel Transwell assay. As anticipated, the migratory capacity of RKO and HCT-116 cells was reduced by PSMA2 silencing (**Figures 4C, D**). Thus, these data demonstrated that the migratory and invasive capacities of CRC cells were enhanced by PSMA2.

### PSMA2 Was a Target of miR-132

**Figure 5:**
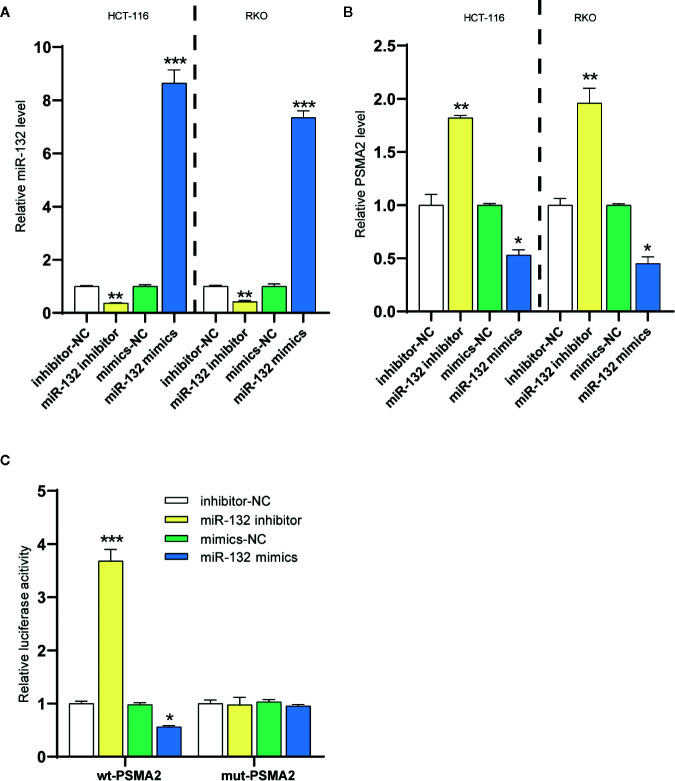
PSMA2 was a target of miR-132. **(A)** The transfection efficiency of miR-132 overexpression or knockdown was determined in CRC using RT-PCR. **(B)** The expression level of PSMA2 was determined after miR-132 overexpression or knockdown. **(C)** Dual luciferase assay was applied to determine the interaction among miR-132 and PSMA2. *P < 0.05, **P < 0.01, ***P < 0.001.

In the study, HumanTargetScan was used to predict PSMA2 as a potential target of miR-132 from biological information (http://www.targetscan.org/cgi-bin/targetscan/vert_71/). To further study the interrelationship between miR-132 and PSMA2, miR-132 was transfected into two CRC cell lines, RKO and HCT-116. The expression of miR-132 was validated by qPCR (**Figure 5A**). PSMA2 expression in RKO and HCT-116 cells with miR-132-overexpression or knockdown was evaluated by qPCR (**Figure 5B**). As shown in **Figure 5B**, compared to the corresponding control cells, miR-132-overexpressing cells had obviously lower expression of PSMA2, while miR-132-knockdown cells had significantly higher expression of PSMA2. These findings showed the correlation between miR-132 and PSMA2 in CRC cell lines.

The online prediction indicated that miR-132 could bind to PSMA2. Then, a wt-PSMA2 plasmid and a mut-PSMA2 plasmid with a 5 bp mutation in the predicted miR-132 binding site were constructed. The abovementioned plasmids and the miR-132 mimic or miR-132 inhibitor were cotransfected into HCT-116 cells to determine luciferase activity. **Figure 5C** showed that the miR-132 mimic and miR-132 inhibitor greatly decreased and increased, respectively, the luciferase activity of wt-PSMA2. More importantly, the miR-132 binding site mutation reversed the miR-132 inhibitor- or miR-132 mimic-induced changes in luciferase activity. These results revealed that PSMA2 was a target of miR-132.

### MiR-132 Suppressed Colorectal Cancer Cell Proliferation

**Figure 6:**
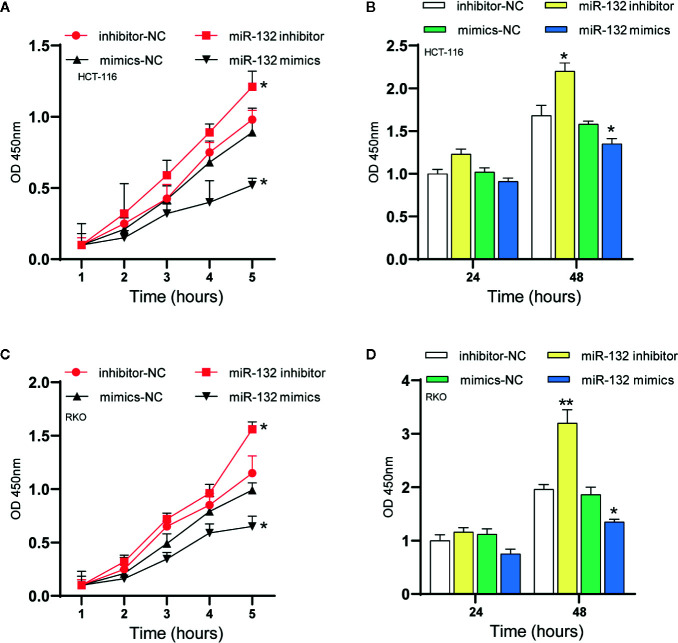
MiR-132 suppresses CRC cell proliferation. **(A, B)** The HCT-116 cell viability after miR-132 overexpression or knockdown was detected using CCK-8 assay. **(C, D)** The RKO cell viability after miR-132 overexpression or knockdown was detected using CCK-8 assay. *P < 0.05, **P < 0.01.

A series of reports stated that miRNAs were critical modulators of tumor cell proliferation (35, 36). MiR-132 is a new candidate due to its inhibitory activity on cancer cells. Here, proliferation assays were applied to identify the specific effect of miR-132 on CRC. The CCK-8 assay revealed that overexpression of miR-132 reduced but miR-132 inhibition improved RKO and HCT-116 cell viability (**Figure 6**).

### PSMA2 Was Up-Regulated and miR-132 Was Down-Regulated in Colorectal Cancer Samples

**Figure 7:**
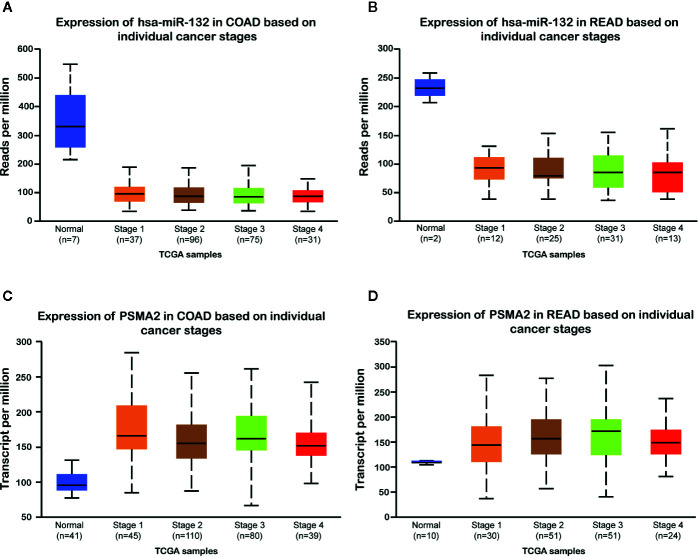
miR-132 was down-regulated and PSMA2 was up-regulated in CRC samples. **(A, B)** compared to normal tissues, both colon adenocarcinoma (COAD) and rectum adenocarcinoma (READ) tissues lowly expressed miR-132 levels. **(C, D)** PSMA2 was significantly up-regulated in both COAD and READ samples.

**Figure 8:**
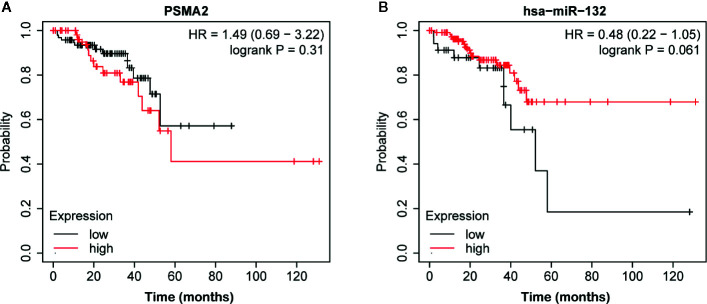
The correlation between miR-132 or PSMA2 levels and overall survival time in CRC. **(A)** No significant correlation between PSMA2 expression and survival time was observed in CRC. **(B)** CRC patients with higher expression of MIR-132 displayed longer overall survival time.

Then, we evaluated the expression of miR-132 and PSMA2 in CRC using the TCGA dataset. Our data showed that compared to normal tissues, both colon adenocarcinoma (COAD) and rectum adenocarcinoma (READ) tissues expressed low levels of miR-132 (**Figure 7**). However, we found that PSMA2 was significantly up-regulated in both COAD and READ samples. Next, Kaplan–Meier survival analysis was performed to evaluate the correlation between PSMA2 and miR-132 expression and overall survival time. The results demonstrated that CRC patients with higher expression of MIR-132 had longer overall survival times (**Figure 8B**); however, no significant correlation between PSMA2 expression and survival time was observed in CRC (**Figure 8A**).

## Discussion

TAMs engender a good metastatic microenvironment and are key determinants of the effectiveness of anticancer strategies (37). TAMs have been revealed to have crucial regulatory roles in CRC progression. For instance, CRC metastasis mediated by mesenchymal circulating tumor cells requires crosstalk between cancer cells and TAMs (38). TAMs in CRC patients are associated with expansion of the microvascular bed. CD204-positive TAMs are related to the malignant transformation of colorectal adenomas (39). TAMs have been indicated to be a prognostic and predictive biomarker of stage II colon cancer in patients receiving adjuvant chemotherapy (40). M2-like macrophages promote the invasion of colon cancer cells *via* matrix metalloproteinases (41). However, the mechanisms by which TAMs affect CRC progression remain largely unknown. Here, 457 TAM-modulated genes were identified. A total of 344 genes were up-regulated, and 113 genes were down-regulated. Bioinformatic analysis revealed that these TAM-associated genes were related to the regulation of macromolecule metabolic processes, apoptotic processes, cell death and programmed cell death regulation and the response to stress. To clarify the interactions among these proteins, we constructed a PPI network. We identified 15 key regulators, including VEGFA, FN1, JUN, CDH1, MAPK8, and FOS, in CRC. Among these genes, we focused on PSMA2 and performed loss-of-function assays to evaluate the potential functions of PSMA2 in CRC.

The biophysical and biochemical clues of tumor-associated extracellular matrix can affect the characteristics of cancer and are therefore essential for malignant tumors. CRC is the most common malignant tumor of the digestive system, and the extracellular matrix organization may affect the tumor characteristics of CRC. A number of studies have shown that ECM–receptor interaction may be related to the occurrence and development of a variety of cancers, including breast cancer, atrial fibrillation, gastric cancer and bladder cancer. Many CRC-related genes studied by previous studies are also enriched in ECM-receptor interaction. Consistent with this article, ECM–receptor interaction might play an important role in CRC. Focal adhesion plays an important role in tumor invasion and metastasis, and can regulate cell function in CRC. The PI3K/AKT-signal pathway is one of the most frequently activated signal-transduction pathways in cancer. It has been reported that the molecular switch in the PI3K–AKT signaling pathway can be used as a potential target for the treatment of CRC.

Previous studies have shown that the expression of VEGFA in human CRC tissues is often down-regulated, miR-150-5p, miR-1249, and microRNA 452 can all regulate the expression of VEGFA and promote the progression of colorectal cancer. FN1 is involved in the process of cell adhesion and migration, and involves various biochemical processes. The up-regulation of FN1 in CRC tissue is a prognostic factor and potential target for CRC treatment. Studies have found that the oncogene transcription factor Jun can inhibit the transcription of miR-22 and play a key role in the progression of CRC. Somatic inactivation of CDH1 is a common early event, and germline mutations can lead to early-onset CRC. MAPK8 accelerates cell proliferation and inhibits the apoptosis of glioblastoma cells. The FOS gene is located on human chromosome 14q21–31 and encodes the nuclear oncoprotein c-Fos. The rs7101 and rs1063169 polymorphisms in the non-coding region of FOS are related to the risk of CRC and the occurrence of CRC. CXCL8 and its receptor are related to the development of various tumor types, especially CRC. The putative tumor suppressor gene EGR1 is transferred in CRC by its code mutations, showing the role in tumorigenesis. Long non-coding RNA CRNDE promotes the proliferation of colorectal cancer cells through epigenetic silencing of CDKN1A expression. The down-regulation of PLK1 is one of the potential mechanisms of the anti-cancer effect of dietary fiber-derived butyrate in CRC. HSPA5 regulates ferroptotic cell death in cancer cells. ITGB1mRNA levels in the recurrence group of CRC patients are up-regulated, which is a potential predictor of CRC recurrence and treatment targets, and predicts the high-risk population of stage II patients. PPARG rs3856806 C>T polymorphism can increase the risk of CRC. ATF3 increases in the serum of CRC patients, which is a potential diagnostic biomarker for CRC patients. COL1A1 can be used as an oncoprotein and can be used as a potential therapeutic target in CRC. We provided more evidence to illustrate the mechanisms in CRC progression.

The functions of PSMA2 in tumorigenesis have been revealed in numerous human cancers. For instance, PAN3-PSMA2 fusion in myelodysplastic syndrome was found to be related to tumorigenesis in acute myeloid leukemia (42). In basal-like breast cancer, knockdown of PSMA2 was found to be associated with both a significant decrease in cell viability and apoptosis induction. In this study, we found that PSMA2 acted as a pivotal node in mediating the effects of TAMs on CRC. Thus, we speculated that PSMA2 was a probable key regulator of CRC tumorigenesis and development. Here, we identified that knockdown of PSMA2 suppressed cell proliferation, migration and invasion, implying that PSMA2 functions as an oncogene in CRC. Moreover, our data indicated that PSMA2 expression was dramatically increased in stage 1, stage 2, stage 3, and stage 4 CRC samples.

MicroRNAs (miRNAs) are small non-coding RNAs that usually suppress messenger RNA (mRNA) translation, reduce mRNA stability, and control genes related to cellular processes, such as inflammation, cell cycle regulation, stress responses, differentiation, apoptosis and migration (43). Moreover, miRNAs participate in the modulation of numerous signaling pathways among most cells, and their dysregulation has been reported to result in cancer occurrence and development. MiR-132 is differentially expressed in a variety of human cancers, including hepatocellular carcinoma, gastric cancer, and breast cancer (44–46). Additionally, miR-132 was shown to participate in the modulation of cell proliferation, apoptosis and metastasis. For instance, miR-132 targets FoxA1 and acts as a tumor suppressor in thyroid cancer (47). MiR-132 inhibits lung cancer cell migration and invasion by preventing USP9X-induced epithelial–mesenchymal transition (48). MiR-132 inhibits ovarian cancer cell proliferation, invasion, and migration by targeting E2F5 (49). MiR-132 impedes the migration and invasion of lung cancer cells by targeting SOX4 (48). MiR-132 causes the metabolic transition of prostate cancer cells by targeting GLUT1 (50). Additionally, miR-132 ablation in CRC is associated with CRC cell invasion and prognosis (51). MiR-132 suppresses the invasion and metastasis of CRC cells by directly targeting ZEB2 (52) and modulates adriamycin resistance in CRC cells by targeting extracellular signal regulated kinase 1 (53). The RT-PCR and luciferase assay results revealed that PSMA2 was one target of miR-132. In addition, our data indicated that miR-132 expression was largely decreased in CRC samples, which was associated with longer survival times of CRC patients, implying that miR-132 is a probable biomarker for CRC.

In addition, our research also initially revealed the influence of PSMA2/miR-132 signaling pathway on the growth of CRC. As far as we know, there is no report about PSMA2/miR-132 in human cancer. We reported for the first time the interaction of PSMA2 and miR-132 in CRC, which provides a new direction for CRC research.

This study has some limitations. It is necessary to collect clinical samples and use clinical samples to confirm the expression of miR-132. In addition, the regulation between miR-132 and macrophages needs further study. Finally, whether macrophages regulate the expression of PSMA2 through miR-132 or other ways will also be explained in the next study.

Taken together, we identified TAM-regulated genes in CRC and constructed a PPI network to reveal the interactions among the selected genes. PSMA2 was identified as a key gene involved in modulating the effects of TAMs on CRC. We showed for the first time that PSMA2 might promote CRC cell proliferation, invasion, and metastasis. In addition, we found that PSMA2 was a target of miR-132, and miR-132 was significantly related to the survival time of CRC patients. miR-132 mimic significantly inhibits the proliferation of CRC cells. The miR-132/PSMA2 axis may be a key mechanism that mediates the development of CRC. These findings are expected to provide a new strategy for CRC treatment.

## Data Availability Statement

The original contributions presented in the study are included in the article/supplementary materials; further inquiries can be directed to the corresponding authors.

## Ethics Statement

The studies involving human participants were reviewed and approved by The Ethics Committee of Minhang Hospital of Fudan University. The patients/participants provided their written informed consent to participate in this study.

## Author Contributions

SW, XS, and LF conceptualized and designed the study. JQ, ZH, and SL developed the methodology. FL collected the samples. SW and XS analyzed and interpreted the study. JQ, ZH, SL, SW, and WW wrote, reviewed, and/or revised the manuscript. All authors contributed to the article and approved the submitted version.

## Funding

This work is supported by Minhang District University Building Project (2017MWDXK03) and Natural Science Foundation of Minhang District Science and Technology Commission (2018MHZ058).

## Conflict of Interest

The authors declare that the research was conducted in the absence of any commercial or financial relationships that could be construed as a potential conflict of interest.
